# Poor Outcomes in Hepatic Amyloidosis: A Report of 2 Cases

**DOI:** 10.1155/2016/7625940

**Published:** 2016-09-28

**Authors:** Brandon Yim, Elizabeth Kertowidjojo, Yue Zhang, Pruthvi Patel

**Affiliations:** ^1^Department of Medicine, Stony Brook University Hospital, Stony Brook, NY, USA; ^2^Department of Pathology, Stony Brook University Hospital, Stony Brook, NY, USA; ^3^Division of Hematology and Oncology, Department of Medicine, Stony Brook University Hospital, Stony Brook, NY, USA; ^4^Division of Gastroenterology, Department of Medicine, Stony Brook University Hospital, Stony Brook, NY, USA

## Abstract

Hepatic amyloidosis is a rare disease entity that results from insoluble amyloid protein deposition in the liver. The disease often presents with vague, nonspecific clinical features. Currently, there is little literature describing treatment outcomes for biopsy-proven hepatic amyloidosis and current treatment guidelines recommend that patients enroll in a clinical trial due to insufficient evidence to suggest an optimal treatment regimen. Here, we present two cases of hepatic amyloidosis at an academic medical center and describe their presentation, treatment, and outcomes. These cases highlight the poor outcomes and difficult management of hepatic amyloidosis. Further understanding and investigation of this rare disease are warranted.

## 1. Introduction

Amyloidosis is a rare disease characterized by the deposition of insoluble, fibril-forming amyloid proteins in the extracellular space of organs, which can result in end-organ dysfunction [1, 2]. Systemic light chain (AL) amyloidosis is the most common form of amyloidosis and results from a plasma cell dyscrasia which produces abnormal amounts of immunoglobulin light chains that can deposit in organs such as the heart, kidney, peripheral nerves, and liver [3]. Liver involvement of amyloidosis usually presents with nonspecific clinical symptoms such as weight loss and fatigue; however, prompt diagnosis is critical as liver-biopsy-proven amyloidosis is known to have a poor prognosis, with a median survival of only 8.5 months [4]. Although established systemic chemotherapy is often used for light chain amyloidosis, current guidelines still recommend that patients enroll in a clinical trial as there is currently insufficient data to suggest an optimal treatment regimen [5]. Therefore, further investigation and reporting of the treatment outcomes are needed. Two biopsy-proven liver amyloidosis cases were identified in the past ten years at Stony Brook University Medical Center. Here we report their presentation, treatment, and outcomes.

## 2. Case  1

A 45-year-old man with a past medical history of hypertension, dyslipidemia, and normocytic anemia presented with a 3-hour history of dull, crampy left lower quadrant abdominal pain. He had an unintentional weight loss of 18 pounds over the course of 5 weeks and also reported generalized fatigue. One month prior to admission, the patient underwent esophagogastroduodenoscopy (EGD), colonoscopy, and video capsule study to evaluate his anemia, all of which were unremarkable. His physical examination was significant for macroglossia and hepatomegaly. Laboratory work revealed a hemoglobin level of 10.8, platelet count of 553, creatinine of 0.8, AST of 119, ALT of 95, total bilirubin of 1.1 (direct bilirubin 0.6), alkaline phosphatase level of 1043, and INR of 1.4. Autoimmune markers and viral hepatitis lab work were negative.

**Figure 1:**
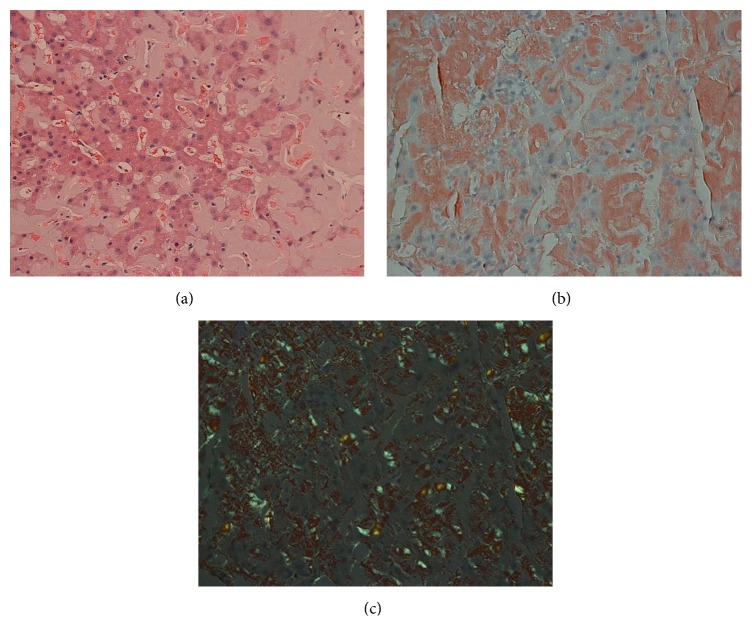
Case  1. Liver biopsy showing architectural distortion with extensive extracellular deposition of eosinophilic material ((a) H&E, ×200), which stains Congo red ((b) Congo red, ×200) and shows green birefringence under polarized light ((c) Congo red, ×200).

A computed tomography (CT) scan of his abdomen revealed hepatosplenomegaly. A transcutaneous ultrasound-guided liver biopsy was performed to further elucidate the etiology of his hepatomegaly. Biopsy tissue showed deposits that stained with Congo red and produced apple-green birefringence under polarized light (Figure 1), characteristic of hepatic amyloidosis.

The patient was referred to the Boston University Amyloid Treatment and Research Program for further management. Work-up revealed a kappa free light chain level of 890 mg/L (normal limits: 3.3–19.4 mg/L) and kappa to lambda free light chain ratio of 270 (normal limits: 5.7–26.3). A bone marrow biopsy was performed and revealed 90 percent plasma cells with kappa predominance of light chain immunoglobulins as well as amyloid deposits, consistent with multiple myeloma. 24-hour urine collection revealed 2.065 g of protein. Troponin I was noted to be 0.028 ng/mL and BNP was noted to be 207 pg/mL. An echocardiogram was performed and revealed an ejection fraction of 66 percent, an intraventricular septal diameter of 13 mm, and normal diastolic function. He was found to have restrictive pulmonary function tests and a chest CT revealing multifocal intralobular septal thickening and patchy nondependent ground glass opacities consistent with diffuse amyloidosis of his lungs.

Ultimately, the patient was diagnosed with AL amyloidosis with associated multiple myeloma with early cardiac, renal, pulmonary, liver, and soft tissue involvement. He underwent 4 cycles of Bortezomib (Velcade) and Dexamethasone and 2 cycles of Revlimid and Dexamethasone with 38 percent reduction of his serum free light chains after three months of treatment, consistent with a very good partial response (VGPR). At this time he also noted improvement of his activity and decreased fatigue. In preparation for an autologous stem cell transplant, he was then switched to weekly CyBorD (Cyclophosphamide 300 mg/m^2^, Bortezomib 1.5 mg/m^2^, and 20 mg Dexamethasone) therapy. Unfortunately, the patient passed away approximately 1 month after the initiation of CyBorD therapy of undocumented reasons. He survived 8 months after his initial diagnosis of amyloidosis.

## 3. Case  2

A 61-year-old man with past medical history of non-insulin dependent diabetes, coronary artery disease, heart failure with preserved ejection fraction, and proteinuria presented with a 3-year history of unintentional weight loss of 14 pounds. He also reported symptoms of early satiety, decreased appetite, fatigue, and dyspnea with exertion. He denied any abdominal pain, night sweats, or fevers. Physical examination revealed a thin man (BMI 19) with hepatomegaly. Laboratory results revealed microcytic anemia with hemoglobin level of 12.8 (MCV 77.7) and an alkaline phosphatase level of 166. Transaminase and bilirubin levels were within normal limits.

A liver ultrasound showed fatty infiltration or fibrosis of the liver. Subsequent MRI of his abdomen revealed hepatomegaly and small branch intraductal papillary mucinous neoplasm (IPMN) of the pancreas. EGD and colonoscopy were significant for Barrett's Esophagus and* Helicobacter pylori* for which he received medical treatment. His alkaline phosphatase levels were persistently elevated within follow-up visits. His viral hepatitis, ANA, anti-mitochondrial antibody, and liver/kidney/microsomal antibody levels were within normal limits. He initially had a high retinyl palmitate vitamin A level of 0.36 mg/L (normal <0.10) from chronic consumption of vitamin A with suspicion of drug-induced liver injury from elevated vitamin A levels; however his levels resolved after suspending vitamin A consumption.

**Figure 2:**
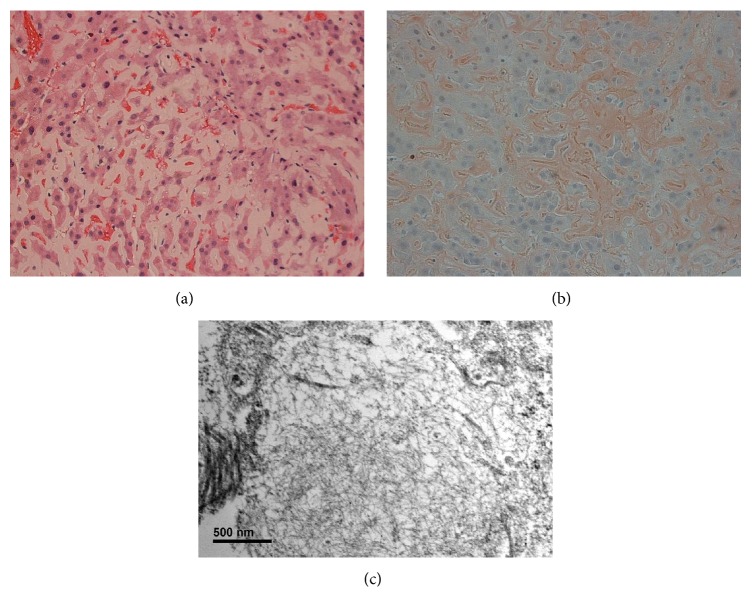
Case  2. Liver biopsy showing architectural distortion with extensive extracellular deposition of amorphous eosinophilic material ((a) H&E, ×200), which stains Congo red ((b) Congo red, ×200). Electron microscopy shows randomly oriented 8 nm diameter ultrastructural fibrils in the extracellular perisinusoidal spaces (c).

An endoscopic ultrasound (EUS) with fine needle biopsy of his liver was performed. The hepatic tissue stained with Congo red and electron microscopy revealed ultrastructural fibrils consistent with amyloidosis (Figure 2). Serum protein electrophoresis (SPE) revealed no apparent monoclonal gammopathy; however, urine protein electrophoresis (UPE) revealed a monoclonal peak in the gamma region. Urine IFE revealed a free lambda light chain. A serum free light chain assay revealed 20.1 kappa light chains (normal: 3.3–19.4) and 88.6 lambda light chains (normal: 5.7–26.3). Bone biopsy revealed monoclonal plasmacytosis of 15–20% with lambda light chain restriction. Troponin T was <0.01. Ntpro BNP measured 927. A cardiac MRI was conducted and was negative for infiltrative myocardial pathology and did not suggest amyloid. Cardiac muscle biopsy was negative for amyloid. Patient was initiated on CyBorD weekly therapy. He received 5 treatments of CyBorD therapy without reduction in his serum free light chains or improvement in his alkaline phosphatase levels. He suffered from cardiac arrest and passed away four months after his original diagnosis.

## 4. Discussion

Although the presentation of hepatic AL amyloidosis has been previously described [4], literature reporting the management and outcomes of these cases is lacking, likely secondary to the rarity and poor prognosis of this disease. Two biopsy-proven liver amyloidosis cases in our institution over the past 10 years were reported here with similar presentations to a previously reported cohort of hepatic amyloidosis patients described by Park et al. [4]. In that cohort of 98 patients, most patients presented with vague, nonspecific symptoms such as weight loss and fatigue, were noted to have hepatomegaly on physical examination, and had elevated alkaline phosphatase levels on presentation [4]. Both of our cases had unintentional weight loss, hepatomegaly, and elevated alkaline phosphatase levels.

Despite receiving treatment regimens consistent with National Comprehensive Cancer Network (NCCN) guidelines [5], the two cases presented here had poor outcomes, surviving only eight and four months after diagnosis for Cases  1 and 2, respectively. Several reasons may explain these outcomes. First, primary hepatic amyloidosis typically presents with advanced disease and is known to have a dismal prognosis with a median survival of only 8.5 months [4]. Both patients had multiple comorbidities that likely contributed to their poor outcomes with cardiac, renal, and pulmonary involvement in Case  1 and coexisting heart failure and proteinuria in Case  2. Second, given the rarity and poor prognosis of hepatic amyloidosis, prompt referral to amyloidosis centers of excellence may have benefited these patients. While Case  1 was referred to the Boston Medical Center's Amyloidosis Program, an established center of excellence, Case  2 received treatment without referral to a designated specialty center. Recent AL amyloidosis management algorithms propose that patients be referred to specialty centers for comprehensive work-up and patient-tailored therapy [6, 7]. Specific testing such as mass spectrometry to confirm amyloid protein composition and genetic testing which has implications on therapy selection can be conducted at these centers to tailor therapy regimens and improve outcomes [8, 9]. Third, careful selection of therapy with close monitoring should occur during the treatment of hepatic amyloidosis cases. In both cases, each patient ultimately was treated with CyBorD therapy, which has been shown to be effective in AL amyloidosis patients with 55 percent of patients projected to survive at 3 years [10]. Unfortunately, both patients in this report died approximately one month after the initiation of CyBorD therapy, raising the possibility of an adverse effect of the CyBorD therapy. In a study by Palladini et al., where AL amyloidosis patients were treated with CyBorD therapy, ten percent of their cohort suffered from severe adverse events, with the most common event being worsening heart failure [10]. Additionally, four of the 230 AL amyloidosis patients treated in that cohort died of sudden cardiac death as was the case in Case  2 of this report [10]. Cyclophosphamide and Bortezomib, primary components of the CyBorD regimen, are both liver-metabolized drugs that could be implicated if these poor outcomes were attributable to adverse effects. Cyclophosphamide, an antineoplastic alkylating agent, has been known to have cardiotoxic side effects such as tachyarrhythmias, complete A-V block, and congestive heart failure with acute symptoms usually presenting 1-2 weeks after administration [11, 12]. Similarly, although described rarely, Bortezomib-induced cardiotoxicity has also been reported in the literature [13]. While Case  1 was noted to have a very good partial response (VGPR) from initial chemotherapy, Case  2 did not respond to therapy despite five treatments with CyBorD therapy. In this disease with poor prognosis, frequent assessment of response to therapy is critical and therapy should immediately be switched if there is no response. In this case, alternative regimens such as high-dose melphalan which is shown to be effective in AL amyloidosis could have been implemented [14]. While we report two poor outcomes of hepatic amyloidosis, it is also worth noting that both patients did not survive long enough to receive stem cell transplant which has been shown to have good outcomes in patients presenting before advanced congestive heart failure [15]. Overall, the poor outcomes of hepatic amyloidosis reported here highlight the importance of prompt diagnosis and comprehensive management of this difficult-to-manage disease. Further investigation and prognostication for primarily liver AL amyloidosis are warranted to improve outcomes for this patient population.

## 5. Conclusion

Amyloidosis of the liver is a rare disease entity that often presents with vague, nonspecific findings and has a poor prognosis. Prompt diagnosis and management are required to improve outcomes for this patient population. Further investigation and research are needed to elucidate the risks and effectiveness of hepatic amyloidosis treatment regimens.
